# Navigating Hereditary Hearing Loss: Pathology of the Inner Ear

**DOI:** 10.3389/fncel.2021.660812

**Published:** 2021-05-20

**Authors:** Teresa Nicolson

**Affiliations:** Department of Otolaryngology, Stanford University, Stanford, CA, United States

**Keywords:** cochlea, organ of corti, hair cells, afferent neurons, stria vascularis, endolymph

## Abstract

Inherited forms of deafness account for a sizable portion of hearing loss among children and adult populations. Many patients with sensorineural deficits have pathological manifestations in the peripheral auditory system, the inner ear. Within the hearing organ, the cochlea, most of the genetic forms of hearing loss involve defects in sensory detection and to some extent, signaling to the brain *via* the auditory cranial nerve. This review focuses on peripheral forms of hereditary hearing loss and how these impairments can be studied in diverse animal models or patient-derived cells with the ultimate goal of using the knowledge gained to understand the underlying biology and treat hearing loss.

## Introduction

Impairment of hearing can be due to several factors including environmental insults, the effects of aging, and hereditary defects. Of these three forms, the most common form is due to aging (Bowl and Dawson, 2019; Keithley, 2020), which can affect the function of the delicate bones and tympanic membrane that mediate the transfer of sound to the hearing organ within the skull, the cochlea. Inside the cochlea, aging typically leads to loss of the sensory receptors for sound known as hair cells owing to a tuft of “hairs” or microvilli-like protrusions at the apical surface. Age-related hearing loss can be greatly exacerbated by a lifelong exposure to damaging levels of sound. If traumatic enough, sound can destroy or weaken the delicate structure of the hair bundle of the sensory cells, leading to permanent damage. In contrast, long term exposure to a less traumatic yet still noisy environment may not adversely affect the hair bundles but may rather cause the disconnection or de-innervation of neurons that receive signals from the hair cells and transmit that information to the brain (Moser and Starr, 2016; Liberman, 2017; Liberman and Kujawa, 2017). Loss of neuronal contacts or synapses with hair cells can eventually result in the death of some of the neurons. Weakened synaptic transmission is usually not apparent in standard hearing tests, but often leads to the inability to hear in noisy environments, also known as the “cocktail party problem.” Aside from loud noise, other environmental insults to the ear include exposure to ototoxic drugs such as aminoglycoside antibiotics that are used to treat life threatening infections or platinum-based drugs used for chemotherapy (Guo et al., 2019). The unintended target of these drugs inside the ear are primarily the hair cells, which can die upon accumulation of the drug inside the hair-cell body (O’Sullivan et al., 2017). The third form of hearing loss involves genetic factors (Korver et al., 2017; Sheffield and Smith, 2019). Hereditary hearing loss is less common than impairments caused by aging and environmental insults, but it is among the most prevalent monogenic diseases in humans.

Hereditary hearing loss is one of the most common sensory deficits in humans affecting one out every 500 newborns (Sheffield and Smith, 2019). The level of impairment can vary widely from profound deafness to mild hearing loss, sometimes varying even among family members with the same mutation. Particular forms of hereditary hearing loss may affect the ability to hear particular frequencies of sound. In addition, the onset of hearing loss can be at the time of birth (congenital), or it can occur in a progressive manner much later in life. To date, hundreds of genes are associated with syndromic (more than the auditory system affected) and non-syndromic (auditory system only) deafness (see text footnote 1).^1^ Of the non-syndromic forms, more than 120 genes have been implicated in hearing loss and the majority of cases involve recessive mutations in which both copies of the gene are mutated. Approximately a third of the cases are dominant, requiring only one copy to be mutated. As with aging, sometimes the middle ear is affected, leading to a loss of conduction of sound. However, the majority of mutations are sensorineural in nature, mainly affecting the inner ear, with many having developmental or functional consequences for hair cells.

Efforts to understand the etiologies associated with hearing loss have been ongoing for several decades. The purpose of this review is to highlight a few recent studies of non-syndromic hereditary hearing loss that illustrate the different types of pathology found in the inner ear. The following studies focus on three different tissue or cell types of the cochlea, namely the stria vascularis, sensory epithelium and the afferent neurons of the spiral ganglion. These studies were also chosen based on the variety of animal models or the use of human-derived cell lines to determine the function of the genes. Due to the inaccessibility of the inner ear in patients, a basic scientific approach with models is necessary to gain a better understanding of the nature of the defects caused by genetic variants that are associated with human hearing loss.

## Generation of the Right Environment for Detecting Sounds: The Stria Vascularis

**FIGURE 1:**
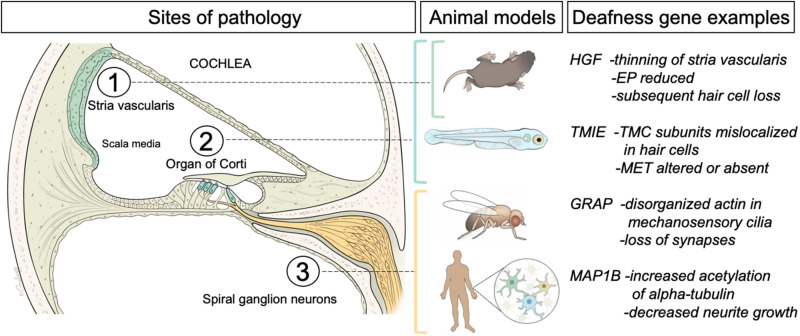
Left panel, schematic of a cross sectional view of the cochlea, the hearing organ of the inner ear. The three regions of interest for this review are indicated. The fluid-filled middle compartment (scala media) contains the stria vascularis, a multilayer of cells (green) that generates the high concentration of potassium ions that are needed for sound detection. The neuroepithelium (organ of Corti) is comprised of sensory hair cells (inner and outer hair cells indicated in blue) embedded in a layer of supporting cells. Auditory hair cells are innervated by afferent or spiral ganglion neurons (yellow) that project to the hindbrain. Middle panel indicates the animal and cell models (*Mus Musculus* mice, *Danio rerio* zebrafish larvae, *Drosophila* flies, and human-derived cells) used for the studies of pathology induced by mutations in the orthologs of four different examples of human deafness genes (right panel). For a more comprehensive list of deafness genes please see the Hereditary Hearing Loss web site (https://hereditaryhearingloss.org/). A brief summary of the findings is included. EP, endocochlear potential; TMC, transmembrane channel-like; MET, mechanotransduction.

An important prerequisite for hair-cell function is the presence of an ionic environment that is conducive to excitation. Unlike other extracellular fluids throughout the body, the fluid inside the scala media of the inner ear is exceedingly rich in potassium ions (Figure 1). The high concentration of potassium ions on the apical side of hair-cell neuroepithelium contrasts greatly to the low concentration of potassium that bathes the basolateral surfaces of hair-cell somas. This striking difference between the two concentrations of potassium generates a dramatic electrochemical potential that facilitates excitation of auditory hair cells. The generation of this unique, high potassium fluid, known as “endolymph,” is carried out by a specialized structure called the stria vascularis (Figure 1, green structure denoted as 1). The tissue of the stria vascularis is comprised of three cell layers. The marginal cells that face the endolymph are positioned on top of a layer of intermediate cells, which are followed by a layer of basal cells. The layers work together to pump and secrete potassium ions from the perilymph to the endolymph [for in-depth reviews, see Wangemann (2006) and Zdebik et al. (2009)]. Accordingly, mutations in ion channels and their regulatory subunits that are expressed in the stria vascularis such as KCNQ1, KCNE1, KCNQ10, and BARTTIN or tight junction molecules such as CLDN11 that are required for the integrity of the cell layers lead to the loss of endocochlear potential and consequently the loss of hearing (Gow et al., 2004; Kitajiri et al., 2004; Rickheit et al., 2008; Chen and Zhao, 2014; Chang et al., 2015; Faridi et al., 2019). In terms of novel gene networks operating in the stria vascularis, recent findings using a single cell RNA-seq approach have revealed expression profiles and further subtypes of cells beyond the marginal, intermediate and basal cells (Gu et al., 2020). In addition, known deafness genes that were thought to act elsewhere such as in the organ of Corti were also found to be expressed in the stria vascularis (Korrapati et al., 2019). The involvement of multiple tissues in causing deafness may mean that robust rescue of hearing using gene therapy will require targeting several cell types.

Although marginal or “dark” cells found in the vestibular inner ear of vertebrates are thought to perform a similar function to the stria vascularis, the electrochemical potential created is substantially lower and defined cell layers are not evident (Wangemann et al., 1995). Furthermore, the structure itself is not present in the hearing end-organs of non-mammalian vertebrates, thus necessitating the study of the pathological consequences of mutations in mammalian model organisms such as the mouse.

A recent example of such a study is illustrated by efforts to understand human hearing loss associated with deletions in the 3′ UTR of hepatocyte growth factor (*HGF*). Despite the name suggesting a specific role in the liver, this extracellular ligand has been implicated in many biological processes involving cell proliferation, survival, and motility. More surprisingly, the only disease in humans associated with mutations in *HGF* is non-syndromic hearing loss. In mice, the knock-out of *Hgf* results in embryonic lethality, whereas a conditional knock-out in the inner ear does indeed result in deafness (Schultz et al., 2009). However, to demonstrate that microdeletions in the non-coding region 3′UTR are causal to the deafness, Morell et al. (2020) engineered a 10 bp deletion in the mouse to mimic the mutation in humans. They found that the stria vascularis was abnormally thin and detached in their mouse model, likely due to a developmental defect wherein neural crest cells fail to migrate and populate the structure. Such a defect is fitting with the role of the HGF protein in cell migration. The failure of cell migration into the stria vascularis was inferred by the reduction of an array of genes expressed by melanocytes, a pigmented neural crest cell that acts as a progenitor source for the intermediate cells. Interestingly, it is this cell type, the intermediate cells, that expresses the receptor for HGF, which is a transmembrane receptor tyrosine kinase known as mesenchymal epithelial transition (MET). Accordingly, a reduction in the endocochlear potential was reported by the authors. Progressive death of hair cells also occurred, presumably due to the changes in the composition of the endolymph.

This example serves as an illustration of the ability to test the causality of specific mutations found in human patients in an animal model such as mice. There is a pressing need for such an approach as genome sequencing of many more affected families forges onward and the putative mutations are identified.

## Detection of Sound: Sensory Hair Cell Receptors

The generation of the high potassium endolymph is key to the function of auditory hair cells. Hair cells rely on the passive movement of potassium from the endolymph to the perilymph *via* their somas for excitation. The mechanism involves mechanoelectrical transduction in which a mechanical stimulus is converted into an electrical signal (Fettiplace, 2017; Ó Maoiléidigh and Ricci, 2019). Hair cells transduce sounds when the wave energy results in a deflection of the apical hair bundle that is exposed to the endolymph. Deflection along the excitatory axis of the hair bundle is thought to cause tension on fine extracellular filaments known as tip links that open mechanotransduction channels (Richardson and Petit, 2019). The passage of potassium through these channels leads to excitation or the depolarization of the cell body. Voltage changes are registered by voltage-gated channels in the basolateral membrane such as Cav1.3a, ultimately leading to the release of neurotransmitter at specialized contacts called ribbon synapses (Moser et al., 2020). Unlike neurons which have all or nothing responses, the excitation of sensory hair cells is a graded response, which is key to passing along information about the intensity of a sound. Information about frequency is based on the mechanics of the cochlea whereby the position itself of the hair cell in the snail-like structure favors the response to a particular pitch of sound (Fettiplace, 2017). For example, hair cells located at the base of the cochlea respond strongly to higher frequencies (in humans up to 20 kHz), whereas hair cells present at the apex are more responsive to lower frequencies (as low as 20 Hz in humans). In the organ of Corti in mammals, hair cells occur in two distinct types: a row of inner hair cells that responds to sound and three rows of outer hair cells that amplify those sounds when needed (Figure 1, structure denoted as 2). Each hair cell is surrounded by supporting cells, which participate in supportive functions such as maintaining structural integrity of the neuroepithelium, clearing potassium and excess neurotransmitter, and generation of extracellular structures that facilitate mechanotransduction [for a comprehensive review see Wan et al. (2013)].

With respect to sensorineural deafness, the vast majority of mutations identified thus far have deleterious effects on hair cells. The list of genes associated with hearing loss affects a wide array of biological processes in hair cells including transcription of genes, organization of the cytoskeleton, mechanotransduction, and synaptic transmission (Hilgert et al., 2009; Müller and Barr-Gillespie, 2015). In mouse models of human deafness, the most common phenotype is the degeneration of hair cells. This outcome is similar to the eventual loss of hair cells induced by defects in the function of the stria vascularis. Thus, examination of earlier stages of development before the onset of degeneration in animal models is often key to understanding the function of particular deafness genes. For example, the architecture of the hair bundle and/or mechanotransduction may be disrupted well before degeneration sets in.

Although many cell types of the cochlea are unique to mammals, the detection of sound by sensory receptors in the inner ear is an ancient sense, and most of the fundamental mechanisms of hair-cell function are highly conserved among vertebrates. For this reason, it is possible to study hair-cell function in a wide range of species. With CRISPR-based gene editing or other genetic methods such as mutagenesis screens, it is also possible to generate animal models of hearing loss outside of rodent models.

As an illustration of (i) using a non-rodent model, the zebrafish, to study human hearing loss, and (ii) the ability to compare the findings with this animal model to that of the mouse model, this review will focus on two recent studies of transmembrane inner ear (*TMIE*). Zebrafish have an inner ear that is anatomically similar to the vestibular portion of human ears. For hearing, they use the saccular end-organ. The sensory hair cells in the zebrafish inner ear rely on many of the same basic components necessary for hearing and balance in humans, and well over a dozen models of human hearing loss have been generated and studied in detail [for comprehensive reviews of studies in zebrafish see Nicolson et al. (1998); Nicolson (2015); Blanco-Sánchez et al. (2017); and Pickett and Raible (2019)].

Previous reports on *TMIE* demonstrated that loss of *TMIE* function resulted in deafness in fish, mice and humans, indicating the importance and conserved function of this gene among vertebrates (Mitchem et al., 2002; Naz et al., 2002; Cho et al., 2006; Shen et al., 2008; Gleason et al., 2009; Zhao et al., 2014). The mechanism, however, was not clear. In zebrafish, the studies of *tmie* were grounded in unbiased forward genetic screens for mutants with balance and hearing defects (Gleason et al., 2009; Pacentine and Nicolson, 2019). In mice, spontaneous mutations in *Tmie* were also identified (Naz et al., 2002; Cho et al., 2006). Initially it was reported that a striking developmental defect in the growth and maturation of hair bundles was observed in *tmie* zebrafish mutants (Shen et al., 2008; Gleason et al., 2009), but this defect was not observed in later studies of hair bundles in either the zebrafish or mouse knock-out mutants (Zhao et al., 2014; Pacentine and Nicolson, 2019; Cunningham et al., 2020). Instead, the main effect of the loss of TMIE protein appeared to be a functional defect resulting in the absence of mechanotransduction (Zhao et al., 2014; Pacentine and Nicolson, 2019; Cunningham et al., 2020). In both fish and mouse mutants, there is a pronounced reduction in the trafficking and localization of a component of the mechanotransduction machinery, the transmembrane channel-like (TMC) TMC1 protein. *TMC1* is a known deafness gene and collective evidence largely supports the idea that the multi-pass transmembrane proteins TMC1 and TMC2 are subunits of the mechanotransduction channel in hair cells [for a review see Corey et al. (2019)]. Mice rely mainly on *Tmc1* at the onset of hearing. In fish where the *tmc2* gene is duplicated, both duplicates (*tmc2a* and *tmc2b*) play a more critical role in hearing (Chen et al., 2020; Smith et al., 2020). Indeed, in *tmie* null mutants, GFP-tagged Tmc2b proteins are not detectable in the hair bundle (Pacentine and Nicolson, 2019). Aside from the role in trafficking of the TMCs to the hair bundles where they are thought to form the pore of the channel, TMIE itself is highly enriched in hair bundles, and is required for the stability of the TMCs at the site of mechanotransduction in both fish and mice (Pacentine and Nicolson, 2019; Cunningham et al., 2020). Therefore, TMIE acts as more than a chaperone for the TMCs, that is, TMIE is also an integral component of the transduction machinery. In *Tmie-/-* mice TMC2 is still present in cochlear hair bundles but inactive without TMIE, however, partial loss-of-function mutations in TMIE can affect TMC2-dependent mechanotransduction channel properties in hair cells (Cunningham et al., 2020). These results suggest that TMIE contributes to the function of the mechanotransduction channel.

The above studies demonstrate that the principal role of TMIE in mechanotransduction is similar among vertebrates. Nevertheless, it also reveals differences that are reflective of having a more flexible array of paralogs or interchangeable components for the mechanotransduction machinery in hair cells. What is common to both animal models is the mislocalization of TMC1, which is essential for hearing in humans. Using a diversity of animal models to explore the basic understanding of how hearing works allows us to identify what is conserved at the core of a biological process such as mechanotransduction and to infer that mutations in the human orthologs will cause similar pathologies in humans.

## Transmission of Auditory Information to the Brain: Afferent Neurons

As mentioned above, hair cells release neurotransmitter at their basolateral surface, which is innervated by afferent neurons. The contacts between hair cells and afferent neurons are referred to as the first order synapses of the ascending auditory system. Auditory afferent neurons have a bipolar-type of morphology with their dendrites or peripheral processes forming the synapses with hair cells and their long axons (central process) projecting out of the bone-encased inner ear into the hindbrain. The fibers of the afferent neurons in the cochlea along with the cell bodies comprising the spiral ganglion are shown in yellow in Figure 1 (denoted as 3). Recent efforts using single cell RNA-seq have identified several subtypes that either belong to the class of spiral ganglion neurons known as Type I that innervate inner hair cells, or a less numerous Type II class that synapses with outer hair cells [for an in depth review see Pavlinkova (2020)]. The tonotopic organization of the cochlea is largely preserved in the projection pattern of the spiral ganglion neurons to their target in the hindbrain, the cochlear nucleus. Thus, information about frequency is also represented spatially at the second order synapse of the auditory system.

As with the stria vascularis and the organ of Corti, hereditary mutations can affect the spiral ganglion neurons and the impairments are known as auditory neuropathies (De Siati et al., 2020). However, there are just a handful of spiral ganglion genes to date that are associated with human hearing loss. These include the deafness genes *DIAPH1*, *TMPRSS3* and *PJVK* (Lynch et al., 1997; Scott et al., 2001; Delmaghani et al., 2006), but as the following examples illustrate, the list is growing.

Using fruit flies to study human deafness genes may seem like a surprising choice but there are remarkable parallels between the mechanisms employed in insect and vertebrate hearing [for reviews see Boekhoff-Falk (2005); Boekhoff-Falk and Eberl (2014); and Hehlert et al. (2020)]. Moreover, similarities exist in the expression patterns of several homologous genes in both the *Drosophila* hearing organ (known as Johnston’s organ) and the vertebrate inner ear. Of the genes required for auditory function in fruit flies, 20% have human orthologs implicated in deafness (Senthilan et al., 2012). For example, like vertebrate ears the developmental genes such as *atonal* and *distalless* are expressed in the auditory organ of *Drosophila*, as are the homologs of known deafness genes such as *myosin VIIa*, *diaphanous*, and *prestin*. The expression of shared genes is indicative that an ancestral mechanosensory cell, if not an ancestral hearing organ, existed in a predecessor that lived before the split of the insect and animal lineages.

A recent study of a novel deafness gene serves as an excellent example of the use of flies to study hereditary hearing loss (Li et al., 2019). In this study, a missense mutation predicted to result in a Q104L substitution in the growth factor receptor-bound adaptor protein (GRAP) was identified in two families. This protein couples small GTPase protein signaling with receptor activation and the authors demonstrated that the gene is expressed in mouse afferent fibers and the sensory organs or “scolopidia” of the fly auditory organ. A conditional knockout of the fly homolog of *GRAP* called downstream of receptor kinase (*drk*) in the antenna harboring the Johnston’s organ caused deficits in gravity sensing and balance in adult flies. At the cellular level, the sensory scolopidia were disorganized, including abnormalities of internal structures such as actin bundles, and there was a loss of synapses formed by scolopidia neurons in the fly brain. To test the whether the Gln104Leu variant was causative, the authors compared rescue of the behavioral defects in the *drk* fly mutant by expressing the wild type human gene and the human Q104L variant and found that the variant form of GRAP was ineffective at rescuing the fly phenotype. The ability to rescue the deficits of an animal model with the corresponding human gene and test newly identified variants is an important and much needed approach for establishing causality of mutations found in the genome of hearing loss patients.

The study of animal models is invaluable for understanding the potential etiology caused by mutations. Nevertheless, with the recent advances in stem cell biology, it is also possible to take cells from patients such as blood cells and reprogram them into cells that resemble those in the auditory system. Cui et al. (2020) used precisely this approach to study the effects of a progressive form of hearing loss that they identified in three families. They focused on one mutation predicted to cause a substitution, S1400G, in the microtubule associated protein 1B (MAP1B). This protein promotes microtubule assembly and neurite extension primarily during development. To assess the effects of the dominant S1400G mutation in auditory neurons, pluripotent stem cells were generated from blood cells collected from hearing-impaired patients and their unaffected family members. A portion of the stem cells were “corrected” using CRISPR gene editing to eliminate the dominant mutation. The stem cells were then coaxed into differentiating into otic sensory neuron-like cells for *in vitro* experiments. The authors found that sensory neural-like cells heterozygous for the S1400G mutation had defects in later steps of differentiation and physiological responses. These defects were accompanied by a decrease in microtubule dynamics. Most of the heterozygous S1400G cells had shorter neurites in contrast to the CRISPR-corrected cells or sibling-derived cells. To further confirm their findings, the authors engineered a mouse model expressing the same dominant mutation. Heterozygous mutant mice displayed moderate yet progressive hearing loss. In addition, primary cultures of the spiral ganglion neurons exhibited similar decreases in neurite length and altered electrophysiological properties.

The above study highlights the utility of reprogramming patient-derived cells into a desired cell type for *in vitro* studies. This approach is an invaluable method for assessing the pathological consequences of a mutation in human cells. Although the technology is in its infancy, another potentially useful method is to generate human organoids of the inner ear. These 3D structures contain cell types that resemble hair cells and the sensory neurons that innervate them (Koehler et al., 2017; Roccio and Edge, 2019). Inner ear organoids can be generated from either human embryonic stem cells or human pluripotent stem cells. Overall, the process requires several weeks and involves manipulation of four signaling pathways to guide cells to adopt multiple cell fates. Perhaps further improvements in the reproducibility of generating 3D cultures will eventually lead to a more routine use of patient-derived cells to generate inner ear organoids, which would presumably create a more physiological environment in which to study a particular mutation.

## Conclusion

In summary, hereditary sensorineural forms of hearing loss primarily affect the tissues and cell types of the inner ear that are vital to sensing sound and transmitting signals to the brain. Our knowledge of the genetics of hearing loss is growing as the pace of identifying novel human mutations associated with impairments in hearing is rapidly increasing (Shearer and Smith, 2015; McDermott et al., 2019). This increase is fueled by the accessibility and affordability of genome sequencing. Furthermore, the time for therapeutic interventions for hearing loss such as gene therapy is also rapidly approaching (Müller and Barr-Gillespie, 2015; Omichi et al., 2019; Taiber and Avraham, 2019; Delmaghani and El-Amraoui, 2020). The need to understand the underlying pathology induced by a mutation, and whether an approach such as gene therapy, or even whether a currently available therapy like cochlear implantation is likely to work requires the type of studies highlighted in this review. The ability to test the causality of new variants discovered *via* genomic sequencing and to establish animal models or study patient derived cells is a key step toward selecting the appropriate therapeutic approaches to ameliorate hearing loss.

The choice of animal or cell model is dictated by several factors. Although the genetic methods in *Drosophila* are highly advanced, the existence of an obvious ortholog of a human deafness gene is not always the case. In addition, extrapolating the pathological defects in fly mutants to potential defects in human patients can be challenging due to the very different structures of the hearing organs in flies. With respect to zebrafish, some deafness genes may be duplicated due to the large-scale duplication of approximately 40% of the genome in teleost fish during evolution. Depending on the expression pattern of the gene duplicates, it may be necessary knock out and analyze two genes instead of one. Also, fish do not have a cochlea; questions about the structures or tissues present only in the mammalian cochlea cannot be addressed. In mice, the need for dissection and cochlear explants to study the cellular defects makes the work more challenging. Generally, research with this particular animal model tends to be more costly and time consuming. Branching out to the use of human stem cells and organoids to study hearing loss is certainly exciting and gaining traction. Whether *in vitro* differentiated cells truly resemble desired cell types or how well the organoids recapitulate the environment of the inner ear is, however, not clear. To date, generating auditory hair cells from stem cells has yet to be achieved. Despite the above shortcomings, animal and cell models have been very valuable for studies of human hearing loss and continuing efforts with these models will undoubtedly yield more insights into the defects and pathology at the molecular, cellular and physiological level.

Aside from helping patients navigate hearing loss, research with animal models and patient-derived cells increases our basic understanding of how the inner ear works. These studies also add fascinating insights of how this remarkable sensory organ evolved and they provide clues about strategies that are employed to suit the auditory needs of a particular animal. On the whole, the interplay between basic and translational research is a fruitful one and offers hope to patients with hearing loss.

## Author Contributions

The author wrote the manuscript and confirms being the sole contributor of this work and has approved it for publication.

## Conflict of Interest

The author declares that the research was conducted in the absence of any commercial or financial relationships that could be construed as a potential conflict of interest.

## Footnotes

1 http://hereditaryhearingloss.org
